# Pain Experience and Behavior Management in Pediatric Dentistry: A Comparison between Traditional Local Anesthesia and the Wand Computerized Delivery System

**DOI:** 10.1155/2017/7941238

**Published:** 2017-02-15

**Authors:** Annelyse Garret-Bernardin, Tiziana Cantile, Vincenzo D'Antò, Alexandros Galanakis, Gabriel Fauxpoint, Gianmaria Fabrizio Ferrazzano, Sara De Rosa, Giulia Vallogini, Umberto Romeo, Angela Galeotti

**Affiliations:** ^1^Unit of Dentistry, Bambino Gesù Children's Hospital, IRCCS, Rome, Italy; ^2^Clinique Saint Nicolas, 55 allée Charles de Fitte, 31300 Toulouse, France; ^3^Department of Neuroscience, Reproductive and Oral Sciences, Section of Pediatric Dentistry, University of Naples, Federico II, Via Pansini 5, 80131 Naples, Italy; ^4^Department of Oral and Maxillofacial Sciences, “Sapienza” University of Rome, Via Caserta 6, 00196 Rome, Italy

## Abstract

*Aim.* To evaluate the pain experience and behavior during dental injection, using the Wand computerized delivery system versus conventional local anesthesia in children and adolescents.* Methods.* An observational crossover split mouth study was performed on 67 patients (aged 7 to 15 years), requiring local anesthesia for dental treatments in both sides of the dental arch. Patients received both types of injections in two separate appointments, one with the use of a Computer Delivery System (the Wand STA system) and one with the traditional syringe. The following data were recorded: pain rating; changes in heart rate; level of collaboration; patient satisfaction. The data were analyzed using ANOVA for quantitative outcomes and nonparametric analysis (Kruskal–Wallis) for qualitative parameters.* Results.* The use of the Wand system determined significantly lower pain ratings and lower increase of heart rate than the traditional syringe. During injection, the number of patients showing a relaxed behavior was higher with the Wand than with the traditional local anesthesia. The patient level of satisfaction was higher with the Wand compared to the conventional local anesthesia.* Conclusions.* The Wand system may provide a less painful injection when compared to the conventional local anesthesia and it seemed to be better tolerated with respect to a traditional syringe.

## 1. Introduction

In dentistry, the injection of a local anesthetic represents the greatest source of fear and anxiety, especially in children and adolescents, because it is mainly associated with pain and discomfort [1, 2]. Furthermore, severe anxiety and fear may increase pain perception [3, 4].

Although the aim of local anesthesia is to eliminate pain during dental procedures, the fear connected to the needle puncture is frequently considered a reason for not visiting the dentist [4–6].

Grace et al., summarizing the results from other studies, reported that, in different countries (Belfast, Northern Ireland; Helsinki, Finland; Jyväskylä, Finland; Dubai, UAE; Norway; Dunedin, New Zealand; Singapore), among adolescents and young adults, dental phobics represent from 5 to 15% and 11 to 26% have high dental fear and anxiety [7–11].

Colares et al., in a cross-sectional study on 970 children between 5 and 12 years old, found a prevalence of dental fear and anxiety of 14.4% [12]. The strongest fears are associated with injections [13].

Fear and anxiety-related behavior can be a significant impediment to dental care and can negatively influence the patient's global health [5, 14].

In particular, a recent study, investigating the prevalence of clinical consequences of untreated dental caries and its relation to dental fear, showed that children with high dental fear had 2.05 times the risk of untreated caries as compared to children with low fear [15]. Untreated decayed teeth were found in 28% of five-year-olds and in 39% of eight-year-olds in England, Wales, and Northern Ireland [16, 17]. In a Brazilian study on 8- to 10-year-old schoolchildren, untreated dental caries and their clinical consequences exerted a negative impact on the quality of life [18].

Therefore, there is an urgent need to develop techniques that decrease pain during injection, preventing patients from avoiding dental treatment [19].

The devices used to make needle punctures less painful (slow injection, warmed-up local anesthetic, thin needle, and pretreatment with topical anesthetic gel) are not sufficient for certain patients, especially for noncollaborating fearful and anxious children [3].

To address this need, a computerized local anesthetic system, the Wand STA system (Milestone Scientific, Livingston, NJ), has been developed to reduce pain during injections [4, 20, 21]. The Wand STA system is made up of a computer controlled unit and a hand-piece component, allowing delivering the anesthetic solution at a constant pressure and at a slow rate, potentially below the threshold of pain [1, 22, 23].

Using a slow flow, the drops of solution can anesthetize tissues immediately ahead of the needle, resulting in an imperceptible injection [23, 24].

Furthermore, the lightweight, pen-like hand-piece allows a more controlled insertion of the needle, improving patient comfort and decreasing pain perception and, consequently, fear of injection [22]. Using this device, all local anesthesia techniques can be executed (maxillary and mandibular infiltration, mandibular block, intraligamentary, anterior middle superior alveolar injection, and even palatal approach injection that is considered the most painful) [1].

Several investigations have been conducted to evaluate the effectiveness of the Wand STA system compared to conventional local anesthesia in children. In a recent randomized controlled study on one hundred children aged 8–12 years, Mittal et al. found that pain perception was significantly higher during traditional palatal infiltration injection as compared to computerized palatal infiltration, while there was no difference in pain perception during buccal infiltration with both techniques [25].

In a clinical trial on pediatric patients, conducted by using a crossover design, San Martin-Lopez et al. showed that computerized injection device reduced pain perception compared to the traditional syringe during dental anesthetic management [26]. On the contrary, Kandiah and Tahmassebi in a prospective, randomized, parallel, controlled study on children demonstrated that pain experience was not different using the Wand or the conventional technique [27]. The reasons for these divergences could be mainly ascribed to differences in the study design (i.e., crossover, parallel) and to patient's anxiety levels, because high fear can overwhelm any distinctions in pain perception [13].

In light of these considerations, the aim of this crossover split mouth study was to compare pain rating, assessed by Visual Analogue Scale (VAS); level of collaboration, assessed by modified Venham scale; and changes in heart rate and level of satisfaction of the patient during the injection, using the Wand STA system versus the conventional local anesthesia, in children and adolescents.

## 2. Materials and Methods

### 2.1. Ethical Aspect

The study was performed in accordance with the Declaration of Helsinki and it was approved by the local Hospital Authority. A detailed informed written consent form was signed by each patient's parents or guardian, who participated in this study.

### 2.2. Design

An observational crossover split mouth study was performed at the Bambino Gesù Children's Hospital, Division of Dentistry, Rome, Italy, from June 2015 to October 2015.

### 2.3. Study Population

The study population consisted of 67 children and adolescents, aged 7 to 15 years, recruited among patients from the Division of Dentistry and Orthodontics, at the Bambino Gesù Children's Hospital, Rome, Italy. All patients required local anesthesia for dental treatments in both sides of the dental arch. Participants were in good general health, took no medications, and had no contraindications to the use of local anesthetic.

Three expert pediatric dentists participated in the study. They were calibrated for the modified Venham scale measurement and kappa statistic was used to compare each of the three examiners to one examiner used as the gold-standard.

The modified Venham scale is a six-point scale, used to evaluate the patient level of collaboration, ranging from 0 (that means relaxed children) to 5 (that indicates children out of control) [28].

Both types of injection were performed by the same pediatric dentist on each patient in two separate appointments, on a side of the dental arch with the use of a Computer Delivery System (the Wand STA system) and on the other side with the traditional syringe. Type and sequence of administration to each individual were randomly assigned, using a table of random numbers. Patients had to close their eyes during the procedure and the audible sound on the Wand was deactivated; therefore, they did not know which type of local anesthesia was used.

No patients have undergone any previous dental local anesthetic experience, in order to be not influenced by a positive or negative memory.

### 2.4. Injection with the Computerized Delivery System

Each injection was preceded by an application of a spray with lidocaine, Ecocain® (10%), Molteni Dental, Milan, Italy.

The device used was the Wand STA, Wand Dental, Inc. Livingston, NJ, USA.

The system works with a dynamic pressure technology, which enables fluid pressure and flow rate at the needle tip [29]. The preprogrammed injection type was selected on the control unit (STA-intraligamentary injection, speed mode 0.005 mL/sec) and, according to the manufacturer's instruction, a 30-gauge, extra short needle was used to administer the solution. The injection was made with 1.8 mL Optocaine® (mepivacaine hydrochloride: 20 mg/mL; adrenaline: 1 : 100.000), delivered in cartridges. The anesthetic solution was administered into the sulcus of each root of the treated tooth (both buccal and palatal/lingual). The needle was inserted parallel to the long tooth axis, and a drop of local anesthetic solution was immediately deposited before the needle entered the tissue. After 4 to 5 seconds, the needle was apically advanced and an additional volume was administered to each root.

Immediately after the injection, the patients were asked to rate the level of pain perceived during the injection, using a 10-point Visual Analogue Scale (VAS) [30].

Patients were monitored to assess changes in the heart rate. Prior to and after the injection, heart rate was measured using pulse oximeter and recorded.

Modified Venham scale was used to measure the level of collaboration during the injection of local anesthesia.

At the end of the procedure, the patient expressed his/her level of satisfaction on a scale from 1 to 10.

### 2.5. Injection with the Conventional Syringe

The traditional syringe was used on the opposite side of the dental arch. Topical anesthetic (Ecocain spray 10%, Molteni Dental, Milan, Italy) was placed in the area of the injection site. The traditional syringe was SOPIRA® Carpule syringes (Heraeus Kulzer, Hanau, Germany).

The traditional injection was performed according to the standard technique. The injection was made with 1.8 mL Optocaine (mepivacaine hydrochloride: 20 mg/mL; adrenaline: 1 : 100.000), delivered in cartridges and a 30-gauge needle was used.

The child's pain perception was assessed by a VAS. Furthermore, the patient was monitored to measure changes in heart rate prior to and after the injection; the modified Venham scale was used to assess the level of collaboration and, at the end of the procedure, the patient expressed his/her level of satisfaction on a scale from 1 to 10.

### 2.6. Data Collection

A structured form was designed to collect information regarding

1. patient's age;
2. gender;
3. tooth location;
4. type of dental treatment (conservative treatment or extraction);
5. score on VAS;
6. heart rate before and after the injection;
7. score on modified Venham scale during injection;
8. level of satisfaction of the patient (scale from 1 to 10).

### 2.7. Statistical Analysis

The results were analyzed in crossover by comparing intrapatient differences to zero using ANOVA for quantitative criteria: pain (VAS during anesthesia) and cardiac frequency (difference between pre- and postanesthesia cardiac frequency) with adjustment on sequence of techniques and type of treatments (conservative treatment or extraction) and nonparametric analysis (Kruskal–Wallis) for qualitative criteria: modified Venham scale and patient satisfaction. The data were analyzed by using the Statistical Analysis Software (SAS) 9.1 for Windows.

## 3. Results

### 3.1. Calibrated Professionals Evaluation

The kappa statistic comparing each of the 3 examiners to the gold-standard examiner yielded scores of 0.87, 0.78, and 0.90, respectively. The kappa was 0.85 when comparing examiners 1 and 2, 0.79 when comparing examiners 2 and 3, and 0.82 when comparing examiners 1 and 3.

### 3.2. Population Description

This study included 67 children and adolescents, 29 girls and 38 boys, aged 7 to 15 years (mean = 9.37 years, SD = 2.04).

### 3.3. Pain Assessment

38 of the 67 patients found the injection with the traditional syringe to be more painful than the injection with the Wand, while 12 found the injection with the Wand to be more painful than the injection with the traditional syringe.

**Figure 1:**
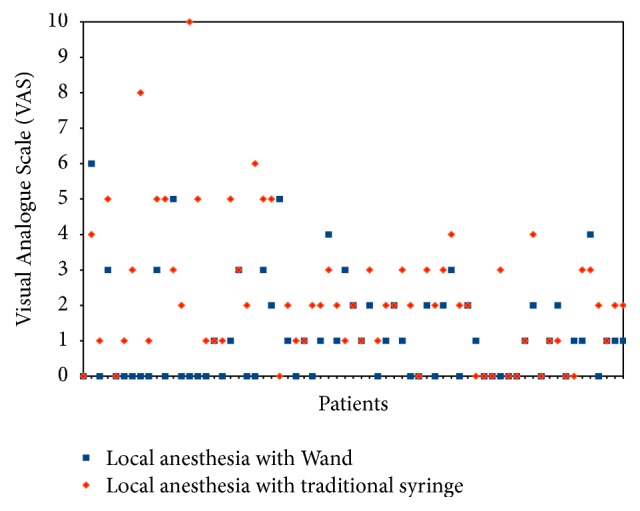
Score on Visual Analogue Scale (VAS) during local anesthesia. 38 of the 67 patients found the injection with the traditional syringe (red) to be more painful than the injection with the Wand (blue), while 12 found the injection with the Wand to be more painful than the injection with the traditional syringe.

Pain during local anesthesia is showed in Figure 1. Concerning pain felt by each patient during both types of local anesthesia, there was no significant difference between boys and girls (*p* ranging from 0.26 to 0.86): mean of VAS for girls was 1.24 during injection with the Wand and 1.91 during injection with the traditional syringe, mean of VAS for boys was 1.26 during injection with the Wand and 2.32 during injection with the traditional syringe.

**Figure 2:**
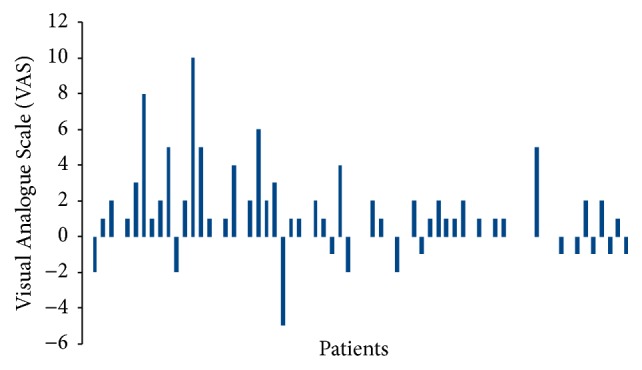
Variation in Visual Analogue Scale (VAS) between Wand and traditional local anesthesia. There was a significant mean reduction of 1.09 VAS point (median 1) with the Wand compared with traditional syringe (*p* = 0.0003).

Figure 2 shows the difference in pain sensation between traditional and Wand techniques.

There was a significant mean reduction of 1.09 VAS point (median 1) with the Wand compared with traditional syringe (*p* = 0.0003). Treatment sequence (*p* = 0.39) and type of treatment (conservative treatment or extraction) (*p* = 0.54) had no significant effect.

### 3.4. Heart Rate Evaluation

Overall mean heart rate was 88 beats per minute before local anesthesia and 93 beats per minute after local anesthesia.

**Figure 3:**
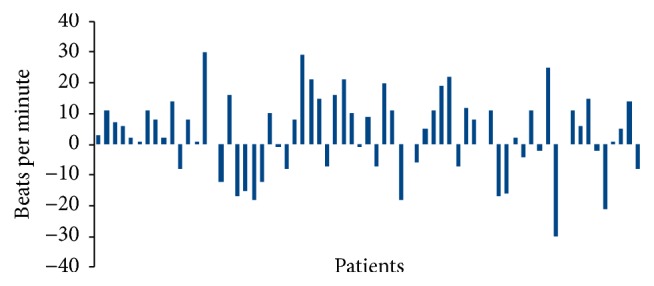
Variation in cardiac rate between Wand and traditional local anesthesia. 41 of the 67 patients had higher rate after injection with the traditional syringe than with the Wand, while 22 showed opposite results.

Figure 3 shows changing in cardiac rate for each patient during both types of local anesthesia.

41 of the 67 patients had higher increase in heart rate after injection with the traditional syringe than with the Wand, while 22 showed opposite results.

There was a significant mean reduction of 3.4 beats per minute (median 5) with the Wand compared with traditional syringe (*p* = 0.028). Treatment sequence (*p* = 0.09) and type of treatment (*p* = 0.94) had no significant effect.

### 3.5. Level of Collaboration Assessment

With the majority of the patients having a modified Venham score of 0, median of modified Venham score was 0 with both techniques. This determined a median intrapatient difference of 0 between both techniques excluding nonparametric comparison.

**Figure 4:**
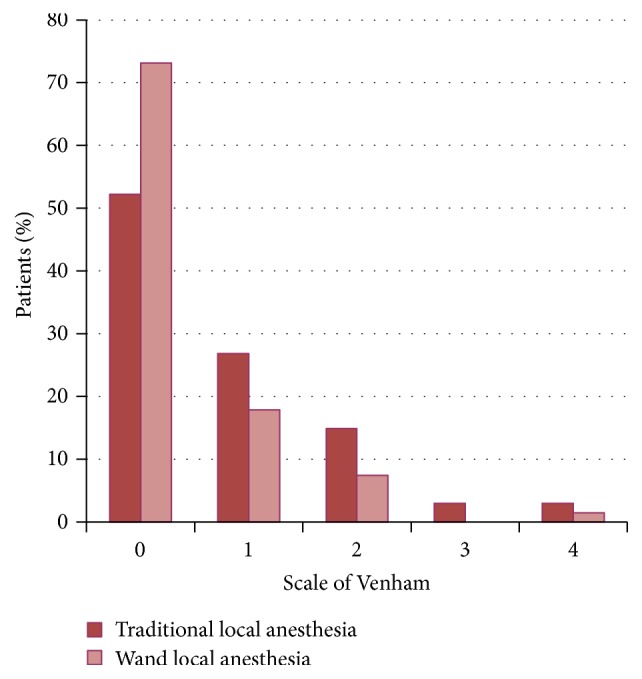
Score on modified Venham scale during local anesthesia. With the Wand methodology more patients have a modified Venham of zero (*p* = 0.019) than with the traditional local anesthesia.

The Fisher exact test demonstrated that with the Wand methodology more patients have a modified Venham of zero (*p* = 0.019) than with the traditional local anesthesia (Figure 4).

### 3.6. Patients Level of Satisfaction Evaluation

Median satisfaction level was 9 with both techniques.

**Figure 5:**
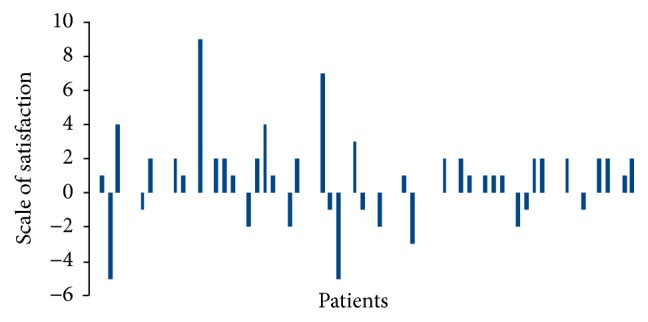
Patients level of satisfaction. 29 patients showed a higher satisfaction after Wand anesthesia, while 12 had a higher satisfaction after traditional anesthesia.

There was a significant mean reduction of 1.09 points on the scale of patient satisfaction with the traditional local anesthesia compared to the Wand system (*p* = 0.0003) (Figure 5).

Treatment sequence (*p* = 0.58) and type of treatment (*p* = 0.89) had no significant effect.

## 4. Discussions

The Wand STA system can be useful in several branches of dentistry, such as pediatric dentistry, restorative dentistry, endodontics, periodontology, and oral surgery [24]. In particular, in the present study, its effectiveness was evaluated on a group of pediatric patients, since children and adolescent's collaboration during dental procedure is the most difficult aspect of patient management, being an interference with quality of care [4].

The analysis of the obtained results revealed that the use of the Wand delivery system in children and adolescents determined lower pain perception and it was generally better accepted than conventional local anesthesia.

Regarding the assessment of pain perception, VAS score was used, because it was easy to understand, even for children.

Our results, showing lower pain perception using the Wand system with respect to conventional local anesthesia, were in agreement with other authors. In particular, Langthasa et al. suggested that the computerized system of anesthetic injection resulted in significantly less pain perception when compared with the same children who experienced a traditional injection by the conventional syringe [31]. Furthermore, San Martin-Lopez et al., in a crossover study, showed that computerized injection device reduced pain perception compared to the traditional syringe during the dental anesthetic management [26]. In contrast, Tahmassebi et al., comparing the sensation of pain when injections were given using the Wand system and a conventional technique in preschool and school age children, found no statistically significant differences [32]. These contrasting results could be explained considering that, in the study of Tahmassebi et al., children were randomly assigned to either a treatment (the Wand) or control (conventional local anesthesia technique) group. In that way, each child did not experience pain sensation due to both techniques [32].

Concerning the assessment of heart rate, in our study 41 of the 67 patients had higher increase in heart rate after injection with the traditional syringe than with the Wand. San Martin-Lopez et al. obtained similar results, finding a difference in the heart rate between the computerized and conventional techniques [26]. These results could be due to the effects of anxiety and pain resulting in increased heart rate [33].

Evaluating the level of collaboration, during injection, the number of patients with a modified Venham score of 0 (that means a relaxed child) was higher with the Wand than with the traditional local anesthesia. Similarly, Gibson et al. reported that fewer children exhibited disruptive behavior during palatal injection with the computer-assisted system compared to a conventional syringe [34]. Also, another investigation showed that children displayed better behavior during injection when they received local anesthesia with the Wand than they did when the conventional supraperiosteal buccal infiltration was used [35].

Furthermore, despite median satisfaction level being 9 with both techniques, the patient level of satisfaction was higher with the Wand compared to the traditional local anesthesia. Apart from being less painful, the Wand system could be considered more satisfying for patients, reducing the numbing of soft tissues and avoiding postoperative self-inflicted injuries (tongue or lip biting) [24].

In addition, although not included in the outcomes, it should be highlighted that in the present study both conventional local anesthesia and the Wand computerized delivery system, performed by experienced pediatric dentists, were effective in guaranteeing a painless dental procedure.

Finally, a limitation of the study was the small number of patients. Further studies are required, involving children younger than 7 years old, in order to evaluate the effectiveness of the Wand system on a large scale and even in precooperative children.

## 5. Conclusions

In pediatric dentistry there has been a continual effort to ensure a painless dental care, maximizing comfort, cooperation, and compliance. In view of the obtained results, it may be concluded that the Wand computerized delivery system can provide less painful injections when compared to the conventional local anesthesia in pediatric patients and it seemed to be better tolerated with respect to a traditional syringe. Further studies should be performed to support and emphasize these findings in order to include the Wand system in routine dental practice.
